# The Association Between Single-Child Status and Risk of Abdominal Obesity: Result From a Cross-Sectional Study of China

**DOI:** 10.3389/fped.2021.697047

**Published:** 2021-08-19

**Authors:** Di Gao, Yanhui Li, Zhaogeng Yang, Ying Ma, Manman Chen, Yanhui Dong, Zhiyong Zou, Jun Ma

**Affiliations:** ^1^Institute of Child and Adolescent Health, School of Public Health, Peking University, Beijing, China; ^2^Key Laboratory of Reproductive Health, National Health Commission of the People's Republic of China, Beijing, China

**Keywords:** single child, one-child policy, abdominal obesity, children, obesity

## Abstract

**Background:** Obesity has become a serious problem threatening the health of children and adolescents, and China's one-child policy has affected family structure and parenting practice, which may result in several adverse health outcomes. The present study aims to investigate the association between single-child status and the risk of abdominal obesity in Chinese adolescents and also to compare the differences in the risk of unideal energy-related behaviors.

**Methods:** Data were obtained from a school-based cross-sectional survey conducted in seven provinces of China, in 2012. A total of 31,291 students aged 7–17 years were recruited in this study. Anthropometric measurements were conducted to assess height and waist circumference, and questionnaires were used to obtain information of single-child status, parental educational attainment, parental weight status, and offspring energy-related behaviors. Multivariate logistic regression models were used to estimate the odds ratio (OR) and 95% confidence intervals (95% CI) of single-child status and odds of childhood abdominal obesity and energy-related behaviors.

**Results:** The prevalence of abdominal obesity was 18.2% in single children, which was higher than that of non-single children (13.7%). The prevalence was also higher in single children in different sex and residence subgroups. Logistic regression models showed that single children had 1.33 times (OR: 1.33, 95% CI: 1.24–1.43, *P* < 0.001) higher odds of abdominal obesity compared to non-single children. Single children had 1.08 times higher odds of physical inactivity (OR: 1.08, 95% CI: 1.03–1.14, *P* = 0.004), 1.13 times higher odds of excessive sugar-sweetened beverages (SSBs) consumption (OR: 1.13, 95% CI: 1.05–1.23, *P* = 0.002), and 1.08 times more likely to eat out (OR: 1.08, 95% CI: 1.02–1.13, *P* = 0.006). Those associations were more remarkable in single girls.

**Conclusion:** Being a single child may be associated with a higher odds of childhood abdominal obesity and unhealthy energy-related behaviors. Future interventions and strategies to prevent abdominal obesity should focus on this high-risk population.

## Introduction

Obesity among children and adolescents is one of the emerging public health issues around the world. It is estimated that the global age-standardized prevalence of obesity has increased from 0.9% (0.5–1.3%) in 1975 to 7.8% (6.7–9.1%) in 2016 among boys and from 0.7% (0.4–1.2%) to 5.6% (4.8–6.5%) among girls (1). The rising trends of body mass index (BMI) in children and adolescents have plateaued in many developed countries but have accelerated in east and south of Asia (1). Similarly, the prevalence of overweight and obesity in China has also increased from 4.3% in 1995 to 18.4% in 2014, with a rapid increase in both boys and girls (2, 3). Although China has a lower rate of obesity in children and adolescents (4), their absolute number will be large with the rapid rising trend. There is increasing concern that obese children may be more likely to become obese adults, and an elevated BMI in adolescence may increase the risk of obesity-related disorders in midlife. Furthermore, excessive distribution of central body fat—abdominal obesity—may be highly associated with cardiometabolic risk factors in adulthood (5, 6).

To effectively control the growing trend of obesity, it is important to identify people at risk and implement effective interventions properly. Factors related to obesity include genetic, environmental, and social aspects, of which family structure is considered one of the potential risk factors of abdominal obesity (7). Over the past decades, China has experienced a remarkable transition of family structure. In order to ease the population explosion, the Chinese government launched the “one-child policy” in 1979 (8), which allowed each couple to give birth to only one child. The policy has been implemented for more than 30 years and was successful in population control, which resulted in hosting the largest singleton population in China currently (9).

In recent years, the health effects of single-child status have been of great concern. Previous studies have investigated the risk of childhood obesity in single children. Based on the data from China Health and Nutrition Survey, single children were about four times more likely to be overweight/obese than those having siblings (10). However, other studies have inconsistent conclusions that there was no significant association between single-child status and obesity (11, 12), which implies that there remains to be studied. In addition, all these studies used BMI as an indicator of weight status but paid little attention to the distribution of body fat, especially around the abdomen. BMI is more qualified to describe the accumulation of total body fat, but not visceral fat. Increasing evidence in recent years has supported that abdominal obesity was a stronger risk factor than general obesity for non-communicable disease and was a better predictor for diabetes and metabolic syndrome in childhood (13, 14). Based on the same research database, the previous study showed that anthropometric indices were not effective screening tools for pediatric cardiometabolic risk factors, but the waist-to-height ratio was still one of the indicators that can better predict clustered risk factors in both boys and girls (15). Furthermore, the control of energy balance may be a very important and useful strategy for reducing obesity rates. As its component parts, energy expenditure and energy intake are both currently topics that are given concerns, and we considered them as energy-related behaviors (16). In our study, energy expenditure behaviors included physical activity and screen time (including TV viewing and video game playing, which are considered as the main sedentary activity) (17, 18). Meanwhile, energy intake behaviors included sugar-sweetened beverages (SSBs) consumption (19), fast food consumption (20), and eating out (21), which have been linked to weight gain and obesity among children and adolescents. However, little is known about whether there were significant differences in energy-related behaviors between single children and non-single children.

Therefore, there is an urgent need to assess the association between single-child status and abdominal obesity in children and adolescents. Thus, we used the data from a national representative cross-sectional survey of 7–17-year-old children and adolescents from China. The present study aims to compare the prevalence and odds of abdominal obesity between single children and non-single children and also to assess the association between single-child status and the odds of unideal energy-related behaviors.

## Materials and Methods

### Study Design and Participants

Data in this study comes from the baseline cross-sectional survey of a national multicenter, cluster-controlled trial addressing the intervention of obesity in children and adolescents from seven provinces or cities of China (Hunan, Ningxia, Hunan, Chongqing, Liaoning, Shanghai, and Guangzhou; registration number: NCT02343588). A more detailed description of the study design and conduct can be assessed elsewhere (22). Briefly, a multistage cluster random sampling method was used in determining participants. At first, several regions were randomly selected from each province/city, and 12–16 schools were randomly chosen from each region. In each school, two classes were randomly selected in each grade and the whole class and their parents were invited to participate in this survey; then, those who signed the informed consent were enrolled in this study for physical measurement, blood detection, and questionnaire survey. All survey sites used the same protocol during the implementation process, and all processes of randomization were performed by a staff member who was not involved in the survey. This study was approved by the Medical Ethical Committee of Peking University (IRB No. 00001052-12072).

### Inclusion and Exclusion Criteria

In this study, waist circumference is not a compulsive item that should be measured in everyone. It was required that at least half of the students in each selected class have their waist circumference measured. Finally, a total of 43,132 students aged 7–17 years who had data of waist circumference were recorded in the survey, of which 31,291 remained in the analysis sample for the present study, after excluding participants who did not have valid data on single-child status (*n* = 2,822), residence area (*n* = 703), height (*n* = 1,713), weight (*n* = 1,390), offspring energy-related behaviors (*n* = 3,027), parental weight status (*n* = 1,013), and parental educational attainment (*n* = 1,254).

### Anthropometric Measurements

Anthropometric measurements were conducted by trained investigators in schools according to the standard protocol. Processes for measuring height and waist circumference were similar at all survey sites. Before measurement, participants were required to take off their coat and shoes and wear only underwear. Height was measured with an accuracy of 0.1 cm using a portable stadiometer (model TZG, Jiangyin Hongya Science and Education Equipment Co., Ltd., Jiangyin, China). Waist circumference (WC) was measured with an accuracy of 0.1 cm using a non-elastic tape at the end of a natural breath at the midpoint between the top of the iliac crest and the lower margin of the last palpable rib. Waist-to-height ratio (WHtR) was calculated as WC divided by the height, and a cut-off value of 0.5 was used to define abdominal obesity (23).

### Single-Child Status

Single-child status information was obtained through the parental self-administrated questionnaire by asking “how many children do you have in your family?” If the parents answered that there was only one child in the family, children were put into the “single children” group, and others were put into the “non-single children” group.

### Energy-Related Behaviors in Childhood

Information on the energy-related behaviors in childhood was obtained from children's self-administrated questionnaire, and all participating students completed the questionnaires during school hours, under the instruction of trained investigators or teachers to ensure consistency across all sites. Children were asked about daily behavior habits, including two items for energy expenditure behaviors [moderate to vigorous physical activity (MVPA) and screen time] and three items for energy intake behaviors (SSBs, fast food intake, and eating out).

#### Energy Expenditure Behaviors

For energy expenditure behaviors, children were asked to answer by themselves the questions of physical activity and screen time (hours and minutes).

Information on child's physical activity was recorded by the International Physical Activity Questionnaire-Short Form (IPAQ-SF) (24), which has been widely used in children and adolescents. MVPA was defined as any kind of aerobic activity that increased heart rate and breathing, such as running, basketball, football, swimming, cycling, table tennis, badminton, calisthenics, etc. MVPA was asked by the following questions: “How many days, over the past 7 days, have you done moderate to vigorous physical activity (MVPA)? And on these days that you do MVPA, how much time did you last on average?” Children reported the frequency (days) and duration (hours and minutes) for MVPA over the past 7 days, and the average daily time was calculated as follows: average daily time = (days × duration in each of those days)/7. We defined physical inactivity as MVPA <1 h/day.

Screen time was asked by the following question: “Over the past 7 days, how much time did you spend on watching TV or playing computer or video games on average?” Students reported the duration (hours and minutes) of watching TV or playing computer or video games per day, and the prolonged screen time was defined as ≥2 h/day.

#### Energy Intake Behaviors

For energy intake behaviors, all participants were asked the frequency (days) of SSBs consumption, and the frequency (days) of fast food consumption and eating out. The questions were as follows: “How many days, over the past 7 days, have you drunk sugar-sweetened beverages?,” “How many days, over the past 7 days, have you eaten fast food?,” and “How many days, over the past 7 days, have you eaten out?” Excessive SSBs consumption was defined as >3 days/week; excessive fast food consumption was defined as ≥1 day/week, and eating out was defined as ≥1 day/week.

### Covariates

Parents were asked to report their children's birth weight according to the birth certificate, and children were divided into three categories: low (<2,500 g), normal (2,500–3,999 g), and high (≥4,000 g). Family socioeconomic status was assessed by the parental highest educational attainment and classified into four groups: (1) junior high school or below, (2) senior high school, (3) junior college, and (4) college or above. Paternal and maternal self-reported height (in centimeters) and weight (in kilograms) were collected from parental questionnaires and used to calculate BMI. BMI of 24 and 28 kg/m^2^ were used to define parental overweight and obesity, respectively, according to the criteria recommended by the Working Group on Obesity in China (WGOC) for Chinese adults (25), and parental weight status was divided into “normal,” “overweight,” and “obesity.”

### Statistical Analysis

Descriptive statistics were calculated for all variables. Mean and standard deviation (SD) were presented for continuous variables, and frequency and percentage were reported for categorical variables. Chi-square (χ^2^) tests or independent-sample Student's *t*-tests were performed appropriately to examine the difference in categorical or continuous variates between single children and non-single children. Multivariate logistic regression analyses were performed to estimate the odds ratio (OR) and 95% confidence intervals (95% CI) of abdominal obesity and unideal energy-related behaviors in the single children group compared to the non-single children group. Potential confounders were adjusted in the logistic regression models, with sex, age, and residence adjusted in Model 2 and additional birth weight, parental educational attainment, and parental weight status adjusted in Model 3. All statistical analyses were conducted using SPSS software version 20.0 (Statistics 20.0, SPSS, IBM, Armonk, NY, USA), and a two-sided *P* ≤ 0.05 was considered as statistically significant.

## Results

### Descriptive Characteristics of the Study Population by Single-Child Status

The descriptive characteristics of the study participants by single-child status are presented in Table 1. A total of 31,291 children (21,146 single children) were enrolled in this study, with a mean age of 11.1 ± 3.0 years. The 50.0% (*n* = 15,659) of the participants were boys and 58.4% (*n* = 18,270) lived in urban areas. The proportion of single children was higher in boys and urban participants (*P* < 0.001). Additionally, compared to non-single children, single children were identified with higher proportion of excessive SSBs consumption (12.6 vs. 11.2%), excessive fast food consumption (9.4 vs. 8.2%), and eating out ≥1 day/week (51.1 vs. 45.2%), but lower proportion of physical inactivity (32.2 vs. 34.3%) and prolonged screen time (21.0 vs. 26.2%).

**Table 1:** Descriptive characteristics [mean (SD) or *n* (%)] of the study participants by single-child status.

**Variables**	**Total (*N* = 31,291)**	**Single children (*N* = 21,146)**	**Non-single children (*N* = 10,145)**	***P-*value**
**Sex**, ***n*****(%)**				
Boys	15,659 (50.0)	11,272 (53.3)	4,387 (43.2)	<0.001
Girls	15,632 (50.0)	9,874 (46.7)	5,758 (56.8)	
**Residence area**, ***n*****(%)**				
Urban	18,270 (58.4)	13,076 (61.8)	5,194 (51.2)	<0.001
Rural	13,021 (41.6)	8,070 (38.2)	4,951 (49.8)	
Age, years	11.1 ± 3.0	11.1 ± 3.0	11.2 ± 3.1	<0.001
Height, cm	147.5 ± 15.7	147.8 ± 15.9	146.7 ± 15.3	<0.001
Waist circumference, cm	65.8 ± 10.8	66.3 ± 11.0	64.9 ± 10.2	<0.001
WHtR	0.5 ± 0.1	0.5 ± 0.1	0.4 ± 0.1	<0.001
**Weight status**, ***n*****(%)**				<0.001
Thinness	1,838 (5.9)	1,233 (5.8)	605 (6.0)	
Normal weight	21,517 (68.8)	14,084 (66.6)	7,433 (73.3)	
Overweight	4,237 (13.5)	3,042 (14.4)	1,195 (11.8)	
Obesity	3,699 (11.8)	2,787 (13.2)	912 (9.0)	
**Paternal weight status**, ***n*****(%)**				0.328
Normal	15,796 (50.5)	10,716 (50.7)	5,080 (50.1)	
Overweight	11,953 (38.2)	8,073 (38.2)	3,880 (38.2)	
Obesity	3,542 (11.3)	2,357 (11.1)	1,185 (11.7)	
**Maternal weight status**, ***n*****(%)**				<0.001
Normal	23,850 (76.2)	16,714 (79.0)	7,136 (70.3)	
Overweight	6,092 (19.5)	3,664 (17.3)	2,428 (23.9)	
Obesity	1,349 (4.3)	768 (3.6)	581 (5.7)	
**Parental highest educational attainment**, ***n*****(%)**				<0.001
Junior high school or below	11,186 (35.7)	5,316 (25.1)	5,870 (57.9)	
Senior high school	8,934 (28.6)	6,203 (29.3)	2,731 (26.9)	
Junior college	5,364 (17.1)	4,481 (21.2)	883 (8.7)	
College or above	5,807 (18.6)	5,146 (24.3)	661 (6.5)	
**Birth weight**, ***n*****(%)**				<0.001
<2,500 g	980 (3.1)	574 (2.7)	406 (4.0)	
2,500–3,999 g	27,175 (86.8)	18,602 (88.0)	8,573 (84.5)	
≥4,000 g	3,136 (10.0)	1,970 (9.3)	1,166 (11.5)	
**Energy-related behaviors in children**, ***n*****(%)**				
Physical inactivity	10,290 (32.9)	6,807 (32.2)	3,483 (34.3)	<0.001
Prolonged screen time	7,088 (22.7)	4,434 (21.0)	2,654 (26.2)	<0.001
Excessive SSBs consumption	3,794 (12.1)	2,662 (12.6)	1,132 (11.2)	<0.001
Excessive fast food consumption	2,817 (9.0)	1,987 (9.4)	830 (8.2)	<0.001
Eating out ≥1 day/week	15,350 (49.2)	10,780 (51.1)	4,570 (45.2)	<0.001

*Physical inactivity was defined as MVPA <1 h/day; prolonged screen time was defined as screen time ≥2 h/day, excessive SSBs consumption was defined as >3 days/week; and excessive fast food consumption was defined as ≥1 day/week. WHtR, Waist-to-height ratio; SSBs, sugar-sweetened beverages; MVPA, moderate to vigorous physical activity*.

### The Prevalence of Abdominal Obesity in Single Children and Non-single Children, by Sex and Residence

**Figure 1:**
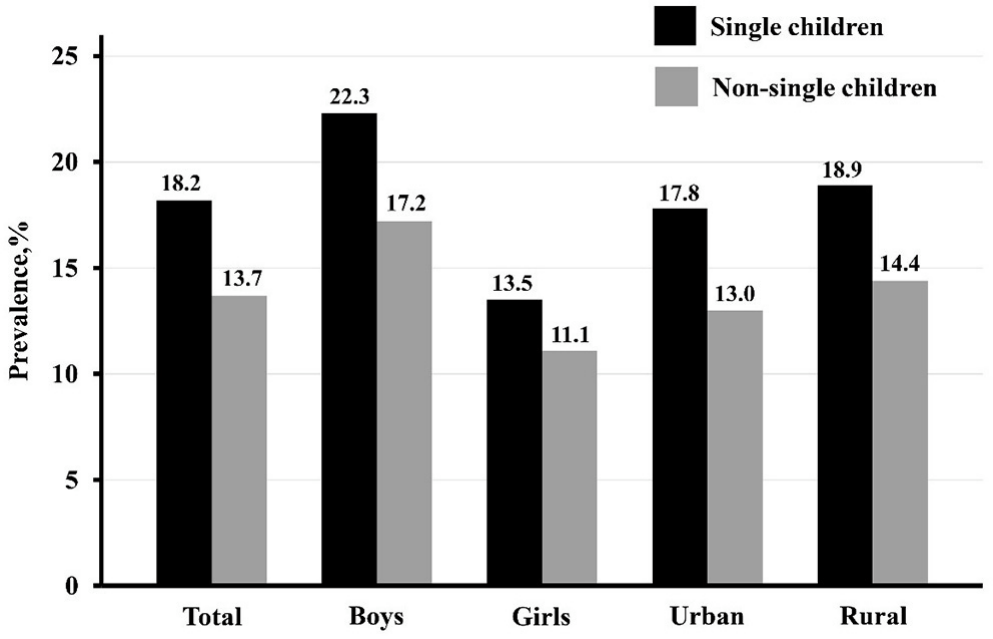
The prevalence of abdominal obesity among single children and non-single children, stratified by sex and residence.

Figure 1 shows the prevalence of abdominal obesity in single children and non-single children, stratified by sex and residence. Overall, about 18.2% of single children were identified as abdominal obesity, compared with 13.7% in non-single children. A higher prevalence of abdominal obesity was observed in single children than non-single children in both sex and residence subgroup participants. Specifically, single boys were observed to have the highest prevalence of abdominal obesity (22.3%), and girls with siblings were observed to have the lowest prevalence of abdominal obesity (11.1%).

### The Association Between Single-Child Status and Odds of Abdominal Obesity and Unideal Energy-Related Behaviors

The associations between single-child status and the odds of abdominal obesity and energy-related behaviors were assessed using multivariate logistic regression models. In total participants (Table 2), single children were estimated to have 1.33 times (OR: 1.33, 95% CI: 1.23–1.43, *P* < 0.001) higher odds of abdominal obesity compared with non-single children. Besides, single-child status was associated with higher odds of physical inactivity (OR: 1.08, 95% CI: 1.03–1.14, *P* = 0.004), excessive SSBs consumption (OR: 1.13, 95% CI: 1.05–1.23, *P* = 0.002), and eating out (OR: 1.08, 95% CI: 1.02–1.13, *P* = 0.006) after adjusted for potential covariates.

**Table 2:** The associations between single-child status (single children vs. non-single children) and odds of abdominal obesity and unideal energy-related behaviors.

	**Model 1**		**Model 2**		**Model 3**	
**Outcome variables**	**OR (95% CI)**	***P*-value**	**OR (95% CI)**	***P*-value**	**OR (95% CI)**	***P*-value**
Abdominal obesity	**1.40 (1.31, 1.50)**	**<0.001**	**1.34 (1.25, 1.43)**	**<0.001**	**1.33 (1.23, 1.43)**	**<0.001**
Physical inactivity	**1.10 (1.05, 1.16)**	**<0.001**	**1.12 (1.07, 1.18)**	**<0.001**	**1.08 (1.03, 1.14)**	**0.004**
Prolonged screen time	**0.75 (0.71, 0.79)**	**<0.001**	**0.75 (0.71, 0.79)**	**<0.001**	0.95 (0.86, 1.02)	0.086
Excessive SSBs consumption	**1.15 (1.07, 1.24)**	**<0.001**	**1.11 (1.03, 1.20)**	**0.006**	**1.13 (1.05, 1.23)**	**0.002**
Excessive fast food consumption	**1.16 (1.07, 1.27)**	**<0.001**	**1.14 (1.04, 1.24)**	**0.004**	1.07 (0.97, 1.17)	0.176
Eating out ≥1 day/week	**1.27 (1.21, 1.33)**	**<0.001**	**1.22 (1.16, 1.28)**	**<0.001**	**1.08 (1.02, 1.13)**	**0.006**

*Bold text indicates a statistically significant difference at P <0.05. Model 1 was a binary regression model. Model 2 adjusted for sex, age, and residence. Model 3 adjusted for all the parameters in Model 2 and additionally adjusted for birth weight, parental highest educational attainment, paternal weight status, and maternal weight status. OR, odds ratios; SSBs, sugar-sweetened beverages*.

### The Association Between Single-Child Status and Odds of Abdominal Obesity and Unideal Energy-Related Behaviors in Different Subgroups

When further analyzed by sex and residence (Table 3), similar ORs were observed in all subgroups. Single-child status was associated with higher odds of physical inactivity, excessive SSBs consumption, excessive fast food consumption, and eating out in girls. Besides, single children from urban area were more likely to consume excessive SSBs and fast food and also more likely to eat out. For single children from rural area, they tend to consume more SSBs and fast food than non-single children.

**Table 3:** The associations between single-child status (single children vs. non-single children) and odds of abdominal obesity and unideal energy-related behaviors by gender and residence.

	**Sex**		**Residence**	
**Outcome variables**	**Boys**	**Girls**	**Urban**	**Rural**
Abdominal obesity	**1.34 (1.22, 1.48)**	**1.35 (1.21, 1.51)**	**1.43 (1.29, 1.58)**	**1.39 (1.26, 1.55)**
Physical inactivity	1.04 (0.96, 1.12)	**1.15 (1.06, 1.24)**	1.03 (0.96, 1.11)	1.02 (0.95, 1.10)
Prolonged screen time	0.96 (0.88, 1.04)	0.91 (0.80, 1.03)	0.93 (0.85, 1.01)	1.03 (0.95, 1.12)
Excessive SSBs consumption	1.08 (0.97, 1.19)	**1.14 (1.02, 1.28)**	**1.28 (1.14, 1.43)**	**1.22 (1.09, 1.36)**
Excessive fast food consumption	0.93 (0.82, 1.06)	**1.20 (1.06, 1.37)**	**1.19 (1.04, 1.34)**	**1.24 (1.07, 1.40)**
Eating out ≥1 day/week	1.01 (0.94, 1.09)	**1.13 (1.05, 1.22)**	**1.07 (1.01, 1.15)**	1.06 (0.98, 1.14)

*Bold text indicates a statistically significant difference at P < 0.05. Model was adjusted for age, birth weight, parental highest educational attainment, paternal weight status, and maternal weight status. OR, odds ratios; SSBs, sugar-sweetened beverages*.

## Discussion

In this national representative cross-sectional study, we investigated the association between single-child status and pediatric abdominal obesity and energy-related behaviors. The results suggested that single children had higher prevalence and increased odds of abdominal obesity and also higher odds of excessive SSBs consumption and eating out, compared with those with siblings.

To our knowledge, there were several studies investigating the relationship between single-child status and the risk of obesity. Yang (11) firstly reported this association using data from the China Health and Nutritional Survey (CHNS) and found that single-child status was not independently associated with childhood overweight, but studies from Hunsberger et al. (26) and Haugaard (7) had demonstrated an elevated risk of overweight in single children from European countries. Similar results have also been reported in the study of Li et al. (27), which investigated 19,487 Chinese children and found that being a single child had 1.29 times higher risk of obesity, and Min et al. (10) found an even much greater risk (OR: 4.5, 95% CI: 1.7–12.4). However, all those studies use body mass index to assess the overall obesity status, and rare concern has been given to the accumulation and distribution of body fat, which was regarded as a better predictor for dyslipidemia (28), sleep apnea (29), and other cardiovascular diseases (30). The present study used waist circumference to assess the association with single-child status, which may provide a stronger prediction of cardiometabolic risk in later life.

Although previous studies had demonstrated the association between single-child status and childhood obesity, the potential mechanism remains unclear. In this study, we found that single children tend to do less moderate to vigorous physical activity, and similar results were also found in previous studies (27). For single children, they may experience higher family expectations and pressure for learning, which might at least partially explain why they spent less time on physical activity and screening (27). As for dietary behaviors, we found that single children are likely to consume more SSBs and fast food, which was consistent with results from previous studies. Hunsberger et al. (26) also found that single children have a higher propensity to consume sugar, and their parents were more likely to support food as a reward, and Irwin (31) found that single children tend to be overfed. As SSBs and fast food are high-energy foods, they may lead to an increased risk of obesity. The single child is the focus of the whole family, which may contribute to the overfeeding and over-favoring of children. Although single children may receive more care and resources from family, nutrition and social environment may lead to these care and resources being converted into weight gain.

Distinctive from other countries around the world, the single-child phenomenon in China was a result of the one-child policy, which was introduced by the Chinese government and conducted as a social or political issue, rather than a parental decision. During the past half-century, the Chinese population control policy has experienced a set of adjustments and modifications, from allowing a second child in rural areas in the late 1980's and in couples who are both single-child in 2000 (11) and finally to ending the one-child policy and promulgating a universal two-child-per-family policy in 2016. In 2021, to actively deal with the population aging and optimize the fertility policy, the Chinese government implemented the policy that one couple can have three children and supporting measures. Even if the family planning restrictions are lifted, there were still plenty of single children in families from China. Our study emphasized that more attention should be paid to the nutritional status and family feeding patterns in single children.

For many developed countries, the population is a low-growth type, with a slow or even negative growth for a long time, and the problem of population aging has appeared. Most of these countries have adopted policies to encourage childbirth to varying degrees, such as Japan, France, and Norway. However, there are still some developing countries that implemented the policy of family planning, such as India. In India, efforts have been made over the years by the government to create a favorable policy environment for family planning, such as promoting contraceptive methods, but the policy is suggestive, which was different from the one-child policy in China. China's one-child policy has become history with the promulgation of the new policy to encourage the birth, but its impact on the health of this generation of children and adolescents still deserves attention, and it also provides a case study for other countries in the world.

### Implications

Our findings have some important implications for public health policies. First, single children in China may be an important target population of obesity intervention in the future, and health education measurements at family and community levels should guide their healthy lifestyles as early as possible. Second, our results will help to predict the future obesity trends in China. With the rapid economic development, urbanization, and nutrition transition, children will continue to be exposed to an increasing obesity environment. Therefore, it is important to assess the impact of the large population of single children on the trend of obesity in China. In addition, although the one-child policy in China has become history, the size of single children is likely to remain large for a long time due to the rapid changes in the demographic structure and the legacy of the 30-year one-child policy. The policy can control the rapid rise of population, improve the overall national quality of population using limited resources, and promote social equality and equity. However, such policy may also cause a series of health problems, such as cardiovascular and metabolic diseases, and psychological problems, so the results in our study suggest such policy may provide a case for other countries, especially in developing countries with rapid population growth and population control measures.

### Limitations

There were several limitations in this study. At first, this study was based on a cross-sectional survey, which prevented us from making inferences about the causal relationship between single-child status and pediatric abdominal obesity. Secondly, information about offspring energy-related behaviors was obtained from simple self-administered questionnaires, rather than validated scale or objective methods, such as a food frequency questionnaire (FFQ) (32) or AHEI score (33) for diet or accelerometer for physical activity, which may result in recall bias. Third, our study included only students aged 7–17 years, and those who dropped out or were in the last year of primary and secondary school (grade 6, 9, and 12) were not contacted. So, our study might be able to include selection bias due to such natural selection of samples. However, because of the large sample size in this study, the results of this study were still credible and valuable. Fourth, there might be measurement bias because of the difference in the measurement of people in different selected centers. However, we tried to reduce the influence of the surveyor as much as possible through repeated measurements by experienced research nurses and trained project members and strict training and quality control according to standard procedures. Further studies based on longitudinal data were needed to understand the underlying mechanisms between single-child status and childhood abdominal obesity and to pay special attention to the intervention programs.

## Conclusion

In conclusion, this study demonstrated that single children had higher prevalence of and increased risk of pediatric abdominal obesity. In addition, we also found that single children tend to do less physical activity and consume more SSBs and fast food, especially for single daughters. Future targeted interventions and strategies to combat obesity should be focused more on this high-risk population. Further studies based on longitudinal data are needed to understand the mechanisms and to develop related intervention programs.

## Data Availability Statement

The raw data supporting the conclusions of this article will be made available by the authors, without undue reservation.

## Ethics Statement

The studies involving human participants were reviewed and approved by Medical Ethical Committee of Peking University. Written informed consent to participate in this study was provided by the participants' legal guardian/next of kin.

## Author Contributions

DG and YL performed the data analysis. DG, ZY, YM, MC, YD, and ZZ interpreted, wrote, and finalized the manuscript. JM participated in reviewing and revising of the manuscript. All authors contributed in the conception and design of this study, read, and approved the final manuscript.

## Conflict of Interest

The authors declare that the research was conducted in the absence of any commercial or financial relationships that could be construed as a potential conflict of interest.

## Publisher's Note

All claims expressed in this article are solely those of the authors and do not necessarily represent those of their affiliated organizations, or those of the publisher, the editors and the reviewers. Any product that may be evaluated in this article, or claim that may be made by its manufacturer, is not guaranteed or endorsed by the publisher.
